# Theoretical characterisation of strand cross-correlation in ChIP-seq

**DOI:** 10.1186/s12859-020-03729-6

**Published:** 2020-09-22

**Authors:** Hayato Anzawa, Hitoshi Yamagata, Kengo Kinoshita

**Affiliations:** 1 Graduate School of Information Sciences, Tohoku University, Sendai, Miyagi, Japan; 2 Advanced Research Laboratory, Canon Medical Systems Corporation, Otawara, Tochigi, Japan; 3 Tohoku Medical Megabank Organization, Sendai, Miyagi, Japan; 4 Institute of Development, Aging and Cancer, Tohoku University, Sendai, Miyagi, Japan

**Keywords:** ChIP-seq, Quality control, Strand cross-correlation, Mappability

## Abstract

**Background:**

Strand cross-correlation profiles are used for both peak calling pre-analysis and quality control (QC) in chromatin immunoprecipitation followed by sequencing (ChIP-seq) analysis. Despite its potential for robust and accurate assessments of signal-to-noise ratio (S/N) because of its peak calling independence, it remains unclear what aspects of quality such strand cross-correlation profiles actually measure.

**Results:**

We introduced a simple model to simulate the mapped read-density of ChIP-seq and then derived the theoretical maximum and minimum of cross-correlation coefficients between strands. The results suggest that the maximum coefficient of typical ChIP-seq samples is directly proportional to the number of total mapped reads and the square of the ratio of signal reads, and inversely proportional to the number of peaks and the length of read-enriched regions. Simulation analysis supported our results and evaluation using 790 ChIP-seq data obtained from the public database demonstrated high consistency between calculated cross-correlation coefficients and estimated coefficients based on the theoretical relations and peak calling results. In addition, we found that the mappability-bias-correction improved sensitivity, enabling differentiation of maximum coefficients from the noise level. Based on these insights, we proposed virtual S/N (VSN), a novel peak call-free metric for S/N assessment. We also developed PyMaSC, a tool to calculate strand cross-correlation and VSN efficiently. VSN achieved most consistent S/N estimation for various ChIP targets and sequencing read depths. Furthermore, we demonstrated that a combination of VSN and pre-existing peak calling results enable the estimation of the numbers of detectable peaks for posterior experiments and assess peak calling results.

**Conclusions:**

We present the first theoretical insights into the strand cross-correlation, and the results reveal the potential and the limitations of strand cross-correlation analysis. Our quality assessment framework using VSN provides peak call-independent QC and will help in the evaluation of peak call analysis in ChIP-seq experiments.

## Background

With the development of next-generation sequencing (NGS) technologies and associated dramatic cost reductions, chromatin immunoprecipitation followed by sequencing (ChIP-seq) has become a major method to obtain genome-wide profiles of DNA-binding elements. Although ChIP-seq application studies have revealed massive biological insights, its experimental complexity and low signal-to-noise ratio (S/N), as compared with other NGS applications, still make ChIP-seq analyses difficult [1, 2]. Furthermore, differences among experimental protocols and subsequent data analyses could extensively bias the final peak calling results [3, 4]. As the sample size per study has increased, as in the cases of ENCODE [5] and ROADMAP [6], the need for a quality control (QC) methodology for ChIP-seq has become more important [7].

Several methods for each step of the downstream analysis have been proposed for ChIP-seq QC [8]. In particular, estimation of the S/N is often performed by focusing on the success of the immunoprecipitation and appraising the number of detectable peaks. Among such methodologies, a common metric to evaluate the S/N of ChIP-seq samples is fraction reads in peaks (FRiP, the ratio between total numbers of reads within and outside the peaks). However, FRiP is highly influenced by the total length of regions called as peaks [7], and that peak call results depend on the total numbers of mapped reads [9]. To make FRiP more comparable, analysts can calculate the normalised FRiP values by down-sampling each sample to a fixed number of mapped reads. Nevertheless, the choice of the number of reads is not a trivial problem: a smaller number of reads is preferred to compare as many samples as possible; on the other hand, the fewer the read depths become, the more peaks become false negatives and the FRiP scores lose sensitivity. Moreover, as peak calling results also depend on the peak calling methods [10] and their parameters, only the FRiP scores calculated under the same peak calling procedure can be compared. Therefore, peak call-independent metrics must be applied to overcome these limitations and achieve robust and accurate ChIP-seq QC.

Strand cross-correlation, which is calculated as correlations between the distribution of depth of forward and reverse reads, coupled with shifting one strand relative to the other, is one of the peak call-free ChIP-seq QC methods [11, 12]. These cross-correlation profiles are typically maximal when the shift size is equal to the mean DNA fragment length, and it is usually assumed that higher maximum values are of better quality in each ChIP-seq experiment. These analyses can be performed before peak call and S/N metrics derived from these methods are stable, relative to the total number of sequenced reads [12].

Strand cross-correlation has been commonly used by peak calling tools to estimate the mean DNA fragment length for single-end sequencing samples [13–16] and the metrics proposed by ENCODE and modENCODE consortia are based on the maximum value of a strand cross-correlation profile. However, another peak corresponds to the sequence read length and this can obstruct accurate estimation of the mean DNA fragment length [17]. To manage these problems, Ramachandran *et al.* introduced two types of strand cross-correlation, the naive cross-correlation (NCC) and the mappability-sensitive cross-correlation (MSCC). They first introduced NCC as Pearson cross-correlation between binarised read density and extended it as MSCC by calculating NCC only among the positions where both forward and corresponding shifted reverse positions were uniquely mappable in a genome (called doubly mappable positions). This binarisation is acceptable, as duplicated reads can be regarded as PCR duplicates and removed in typical ChIP-seq experiments [18]. Moreover, the binarisation approach has some advantages over the Pearson cross-correlation in both theoretical and numerical calculations of the cross-correlation coefficient: read density functions and the following derivation can be simplified, and the calculation can be implemented as bit vectors for computational efficiency. Additionally, MSCC can perform accurate fragment length estimation and subsequent detection of the maximum value.

Despite the potential power of strand cross-correlation profiles for ChIP-seq QC assessment, their theoretical characterisation has yet to be undertaken. In this study, we first derived the theoretical maximum and minimum for NCC and MSCC to obtain theoretical insights. Next, based on these results, we proposed VSN as a novel metric to assess ChIP-seq S/Ns.

## Methods

### Overview

The starting objectives in this study were to: (1) clarify theoretical relations among parameters that influence strand cross-correlations; and (2) evaluate such relations by using actual ChIP-seq data. In the following subsections, we first introduce a model under the binarised read counts to express probabilities of how often reads are mapped to regions around binding elements and the other background regions. Secondly, we describe how the expected values of the maximum and minimum NCC coefficients can be calculated. Finally, we derive the theoretical maximum and minimum and compare these results with NCC and MSCC profiles calculated from simulated and actual ChIP-seq data.

### ChIP-seq read density model

**Fig. 1:**
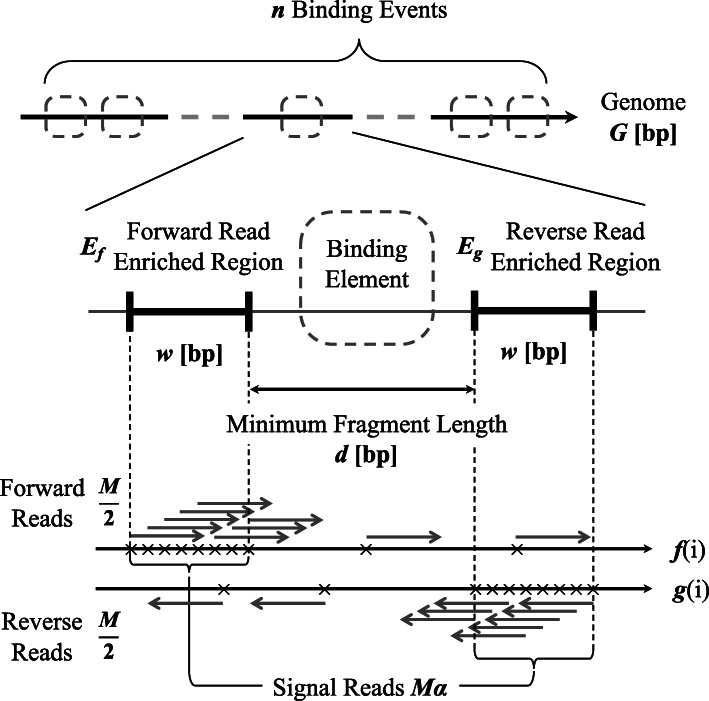
Illustration of the ChIP-seq read density model. The genome whose length is *G* contains *n* binding events. There is a pair of read-enriched regions which belong to *E*_*f*_ or *E*_*g*_ on both sides of each binding element. All the enriched regions have the same width *w* and the forward and the reverse enriched regions in a pair have a distance *d*, which corresponds to the minimum DNA fragment length, across a binding element. We assumed that *M* is the total number of (uniquely) mapped reads in a sample and the numbers of mapped reads into the forward and reverse are same. The total number of the signal reads is denoted *Mα* with the S/N parameter *α*. The total number of the noise reads can be described as *M*(1−*α*)

Various models for ChIP-seq data have been proposed so far. However, these models rely on the control data to consider enrichment [19, 20], the genomic binning-based approach to count reads [21, 22] or empirical read distribution to obtain the probability of read occurrence [23]. To focus on the enrichment (S/N) in a ChIPped dataset and specialise in strand cross-correlation investigation, we propose the following model to express read density distributions in ChIP-Seq (Fig. 1). Here, we assume a genome which has a single chromosome with length *G* but it is readily extended to multiple chromosomes, as shown later, and set *n* as a total number of binding events among the genome. For each binding site, we assume there are the regions where forward and reverse reads are enriched with width *w* bp, and set *d* as a distance between the regions of forward and reverse reads. For $i \in \{1,2,\dots,G\}$, let *f*(*i*) and *g*(*i*), as binary functions, to indicate that one or more reads are mapped, or not, at the position *i* in forward and reverse strands, respectively. Note that these positions used in the functions *f* and *g* represent just the start positions of mapped reads: If a read with the length of *R* is mapped into the forward-strand at positions *i* to *i*+*R*−1, then 1 is assigned only to *f*(*i*). Similarly, if a read is mapped into the reverse-strand of the same positions, 1 is assigned to *g*(*b*+*R*−1).

We assumed that the same number of reads was mapped into both strands:

1 $$ \sum_{i=1}^{G}{f(i)} = \sum_{i=1}^{G}{g(i)} = \frac{M_{\mathrm{u}}}{2},   $$

where *M*_u_ indicates a total number of mapped reads after removal of duplication. An actual total number of mapped reads, (*M*), is sufficiently smaller than *G* in a typical ChIP-seq assay, so the probability of duplicating reads’ positions is quite low. Therefore, we used *M* instead of *M*_u_ except where *M*_u_ was necessary. We divided mapped reads into signal reads and noise among the genome. To formalise this, let *E*_*f*_ and *E*_*g*_ be sets of genomic positions of forward- and reverse-read enriched regions, respectively. In this case, we introduced a mixture parameter, *α*, which indicates a S/N for a ChIP-seq sample, and the following relations are readily obtained:

2 $$\begin{array}{*{20}l} \sum_{i \in E_{f}} f(i) = \sum_{i \in E_{g}} g(i) & = \frac{M}{2}\alpha  \end{array} $$

3 $$\begin{array}{*{20}l} \sum_{i \notin E_{f}} f(i) = \sum_{i \notin E_{g}} g(i) & = \frac{M}{2}(1-\alpha)  \end{array} $$

Equation (2) indicates a total number of signal reads and Eq. (3) indicates a total number of noise reads.

### Expected value of NCC coefficients

The NCC of *f* and *g* at a shift size *x* is formally written and approximately derived as follows [17]:

4 $$ \begin{aligned} \text{NCC}(f, g)(x) & = \frac{1}{G-x}\frac{\sum_{i=1}^{G-x}{(f(i) - \mu_{f})(g(i+x) - \mu_{g})}}{\sqrt{\sigma_{f}\sigma_{g}}} \\ & \approx \frac{\frac{1}{G-x} \bigl(\sum_{i=1}^{G-x}{f(i)g(i+x)} \bigr) - \mu_{f}\mu_{g} }{\sqrt{\sigma_{f}\sigma_{g}}}, \end{aligned}  $$

where *μ*_*f*_ and *μ*_*g*_ are the means of *f* and *g*, and *σ*_*f*_ and *σ*_*g*_ are the variances. We assumed both strands have the same number of mapped reads. Thus:

5 $$\begin{array}{*{20}l} \mu \coloneqq \mu_{f} = \mu_{g} & = \frac{M}{2G}  \end{array} $$

6 $$\begin{array}{*{20}l} \sigma \coloneqq \sigma_{f} = \sigma_{g} & = \mu(1-\mu)  \end{array} $$

The summation in Eq. (4) is equivalent to counting the positions *i* where both *f*(*i*) and *g*(*i*+*x*) are one, which we called *D*_*x*_ and we denote its size as |*D*_*x*_|. If an expected value 〈|*D*_*x*_|〉 is given, the corresponding expected value of NCC can be obtained as:

7 $$ \langle \text{NCC}(f, g)(x) \rangle = \frac{1}{\sigma} (\frac{\langle |D_{x}| \rangle}{G-x} - \mu^{2}).  $$

Next, we estimated |*D*_*x*_| based on the probability that *f*(*i*) and *g*(*i*+*x*) become 1 at the same time. Let *P*_*f*=1_(*i*) be the probability of *f* equals 1 at a position *i*, and let *P*_*g*=1_(*i*) for *g* in a similar manner. Using these probabilities, the expected value of |*D*_*x*_| can be described as:

8 $$ \langle |D_{x}| \rangle = \sum_{i=1}^{G-x}{P_{f=1}(i)P_{g=1}(i+x)}   $$

If we assume uniform distributions, the probabilities of observing a signal read at a position *i* of the forward strand (*P*_S,*f*=1_(*i*)) and to observe a noise read (*P*_N,*f*=1_(*i*)) are:

9 $$\begin{array}{*{20}l} P_{\mathrm{S},f=1}(i) & = \left\{\begin{array}{ll} \frac{M}{2nw}\alpha & \text{if }i \in E_{f} \\ 0 & \text{if }i \not\in E_{f} \end{array}\right.  \end{array} $$

10 $$\begin{array}{*{20}l} P_{\mathrm{N},f=1}(i) & = \frac{M}{2G}(1-\alpha).  \end{array} $$

Therefore, probabilities that *f* becomes 1 at *i* can be written as:

11 $$ \begin{aligned} P_{f=1}(i) & \coloneqq 1 - \big(1 - P_{\mathrm{S},f=1}(i) \big)\big(1 - P_{\mathrm{N},f=1}(i) \big) \\ & = \left\{\begin{array}{ll} p_{\mathrm{S}} & \text{if }i \in E_{f} \\ p_{\mathrm{N}} & \text{if }i \not\in E_{f} \end{array}\right.. \end{aligned}  $$

The *p*_S_ and *p*_N_ will be expanded below. Since all of the parameters appeared in Eq. (9) and (10) are not strand-specific, *P*_*g*=1_(*i*) can be calculated in the same way. As a result, *P*_*g*=1_(*i*) can also be written using *p*_S_ and *p*_N_:

12 $$ P_{g=1}(i) = \left\{\begin{array}{ll} p_{\mathrm{S}} & \text{if }i \in E_{g} \\ p_{\mathrm{N}} & \text{if }i \not\in E_{g} \end{array}\right.   $$

Thus, we can expand *P*_*f*=1_(*i*)*P*_*g*=1_(*i*+*x*) as:

13 $$ P_{f=1}(i)P_{g=1}(i+x) = \left\{\begin{array}{ll} p_{\mathrm{S}}^{2} & \text{if }i \in X_{\text{SS}} \\ p_{\mathrm{S}} p_{\mathrm{N}} & \text{if }i \in X_{\text{SN}} \\ p_{\mathrm{N}} p_{\mathrm{S}} & \text{if }i \in X_{\text{NS}} \\ p_{\mathrm{N}}^{2} & \text{if }i \in X_{\text{NN}} \end{array}\right.,  $$

where

14 $$ \begin{aligned} X_{\text{SS}} & \coloneqq \{i \mid (i \in E_{f}) \land (i+x \in E_{g}) \} \\ X_{\text{SN}} & \coloneqq \{i \mid (i \in E_{f}) \land (i+x \notin E_{g}) \} \\ X_{\text{NS}} & \coloneqq \{i \mid (i \notin E_{f}) \land (i+x \in E_{g}) \} \\ X_{\text{NN}} & \coloneqq \{i \mid (i \notin E_{f}) \land (i+x \notin E_{g}) \} \end{aligned}.  $$

The sizes of these sets can be counted, based on our model, as given by *x*. Finally, we obtained |*D*_*x*_| as:

15 $$ \left\langle |D_{x}| \right\rangle = |X_{\text{SS}}| p_{\mathrm{S}}^{2} + (|X_{\text{SN}}| + |X_{\text{NS}}|) p_{\mathrm{S}} p_{\mathrm{N}} + |X_{\text{NN}}| p_{\mathrm{N}}^{2}   $$

### Unsaturated and saturated cases

In Eqs. (9) and (10), we implicitly assumed that signal and noise reads were not duplicated and each of them had a unique mapped position. In other words, the required conditions to get Eqs. (9) and (10) are:

16 $$\begin{array}{*{20}l} \frac{M}{2}\alpha & \leq nw  \end{array} $$

17 $$\begin{array}{*{20}l} \frac{M}{2}(1 - \alpha) & \leq G  \end{array} $$

In usual mammalian ChIP-seq experiments, *M*<*G* and Eq. (17) will be acceptable. However, small *nw* and/or large *Mα* can break this condition. In other words, $\frac {M}{2}\alpha $ exceeding *nw* means some reads are duplicated in the enriched regions in *f* and *g*. For these cases, other assumptions must be applied; consequently we designated such case as “saturated” and the cases that follow Eqs. (16) and (17) as “unsaturated”. Moreover, based on Eq. (16), we refer to $\frac {M\alpha }{2nw}$ as the “degree of saturation,” which indicates whether a case is saturated or unsaturated, depending on whether the value exceeds one or not. According to Eqs. (9) and (10), *p*_S_ and *p*_N_ for unsaturated cases can be calculated as:

18 $$ \text{Unsaturated Case} \coloneqq \\ \left\{\begin{array}{ll} \mu & = \frac{M}{2G} \\ p_{\mathrm{S}} & = \frac{M}{2G}(1-\alpha) \\ & \quad + \frac{M}{2nw}\alpha \big(1 - \frac{M}{2G}(1-\alpha) \big) \\ p_{\mathrm{N}} & = \frac{M}{2G}(1-\alpha)  \end{array}.\right.  $$

See Additional file 1 for saturated case settings.

### Expected value of MSCC coefficients

MSCC coefficients can be estimated using NCC relations described above. If the distribution of enriched regions and mapped read positions are independent of the double mappable positions, MSCC coefficients are approximately equal to NCC coefficients. See Additional file 1 for details.

### NCC and MSCC calculation in actual ChIP-seq data

#### Mappability calculation

To obtain MSCC profiles, genome mappabilities must be calculated. We used the GEM mappability program [24] to calculate mappability tracks (GEM-indexer build 1.423, GEM-mappability build 1.315 and GEM-2-wig build 1.423) for each sequence read length. Following the ENCODE mappability tracks available at the UCSC Genome Browser [25], up to 2 mismatches were allowed. Mappability profiles generated by GEM were converted into BigWig format files. In this study, as uniquely mappable positions in the genome (i.e. mappability scores equal one) were sufficient to calculate MSCC, we simplified mappability profiles by assigning mappability scores as zero to genomic positions which had mappability less than one.

#### Merging cross-correlation coefficients

In actual ChIP-seq data, coefficients calculated from each chromosome have to be merged into whole-genome coefficients. In this study we applied Fisher’s r-to-z transformation [26] to coefficients and then a weighted average $\hat {z_{\mu }}$ was calculated. An average coefficient was obtained by inversely transforming $\hat {z_{\mu }}$.

#### Implementation

Although we followed the procedures for calculating cross-correlations introduced by [17] and their developed tool, MaSC, to calculate naïve and mappability-sensitive cross-correlation, MaSC is not necessarily appropriate for practical use because of the necessity of file format conversions and unsupporting parallelisation. To enable large-scale cross-correlation analysis, we developed PyMaSC, the Python implemented MaSC algorithm tool. PyMaSC supports BAM and BigWig formats as inputs and multiprocessing for efficiency. PyMaSC is freely available at PyPI (the Python Package Index) and https://github.com/ronin-gw/PyMaSC.

### ChIP-seq data preparation

All ChIP-seq data used in this study were mapped into hg38 using BWA-MEM version 0.7.17 [27] with default parameters and duplicated reads were marked with SAMBLASTER version 0.1.24 [28]. Reads with low mapping quality (< 6) or mapped into alternate or unlocalised contigs were discarded using SAMtools version 1.3.1 [29]. For actual data, we predicted mean fragment lengths manually using MSCC profiles generated by PyMaSC with the genome mappability files we generated. Peak calling analyses were performed by MACS2 version 2.1.0 [13] with corresponding control samples and default parameters except adding --nomodel and --extsize options to specify the predicted fragment length. Additionally, --broad option was applied for broad histone mark samples. Note that *gappedPeak* files were used to count a number of called peaks for broad peak analysis.

### Comparison of strand cross-correlation-based metrics

VSN, which is a new metric we propose later, was compared with the existing strand cross-correlation-based methods: phantompeakqualtools (PPQT) [11] and strand-shift profile (SSP) [12]. Based on each profile, the normalised strand coefficient (NSC) and the relative strand correlation (RSC) were calculated. To obtain an accurate maximum for each profile and focus on the ability of QC assessment, we used manually predicted mean fragment lengths as the standard shift sizes, and then calculated NSCs and RSCs. For VSNs, MSCC profiles yielded by PyMaSC were used.

In this analysis, we did not perform explicit read filtering, such as mapping quality filtering or duplication removal, to clarify the difference in treatment of such reads between the tools.

## Results

### Theoretical maximum and minimum for NCC

The expected value of the NCC coefficient can be calculated from Eqs. (7) and (15) if *p*_S_, *p*_N_, *μ* and the sizes of sets in Eq. (14) are given. Here we clarify the combinations of sizes which give minimum and maximum values of NCC. If there is no overlap between the forward and reverse enriched regions with a shift size of *x*, then *x* will give the minimum of the cross-correlation function. Here we call these *x* as *x*_0_. In this case, the sizes are:

19 $$ \begin{aligned} |X_{\text{SS}}| & = 0 \\ |X_{\text{SN}}| & = nw \\ |X_{\text{NS}}| & = nw \\ |X_{\text{NN}}| & = G - 2nw - x_{0} \end{aligned}   $$

Evidently, the *x*=*d*+*w* maximises the cross-correlation function, and all the pairs of enriched regions exactly overlap:

20 $$ \begin{aligned} |X_{\text{SS}}| & = nw \\ |X_{\text{SN}}| & = 0 \\ |X_{\text{NS}}| & = 0 \\ |X_{\text{NN}}| & = G - nw - (d+w) \end{aligned}   $$

For unsaturated cases, we assumed *p*_S_, *p*_N_ and *μ* at Eq. (18). According to Eq. (19), the minimum cross-correlation coefficient under the unsaturated cases is:

21 $$ \begin{aligned} \text{NCC}(f, g)(x_{0}) & = - \frac{M\alpha \big(M(1-\alpha)^{2} + (G+x_{0})\alpha - 2x_{0} \big)}{(2G-M)(G-x_{0})} \\ & \approx - \frac{M\alpha \big(M(1-\alpha)^{2} + G\alpha \big)}{(2G-M)G} \\ & \approx 0 \end{aligned}   $$

The first approximation requires *x*_0_≪*G* and this relation can be assumed in usual cases. If *M* can be regarded as sufeficiently smaller than *G*, the minimum coefficient approximates 0. The maximum can be calculated from Eq. (20):

22 $$ \begin{aligned} &\text{NCC}(f, g)(d+w) = \frac{M\alpha^{2} \bigl(2G-(1-\alpha)M \bigr)^{2}}{4nw(2G-M)(G-(d+w))} \\ &\quad - \frac{M\alpha \big(M(1-\alpha)^{2} + (G+d+w)\alpha - 2(d+w) \big)}{(2G-M)(G-(d+w))} \\ &\approx \frac{M\alpha^{2} \bigl(2G-(1-\alpha)M \bigr)^{2}}{4nw(2G-M)G} - \frac{M\alpha \big(M(1-\alpha)^{2} + G\alpha \big)}{(2G-M)G} \\ &\approx \frac{M}{2nw}\alpha^{2} \end{aligned}   $$

For saturated cases, the minimum is NCC(*f*,*g*)(*x*_0_)≈0 and maximum is $\text {NCC}(f, g)(d + w) \approx nw / (\frac {M_{\mathrm {u}}}{2}(1-\alpha) + nw)$. See Additional file 1 for complete formulas.

### Evaluation with simulation data

**Fig. 2:**
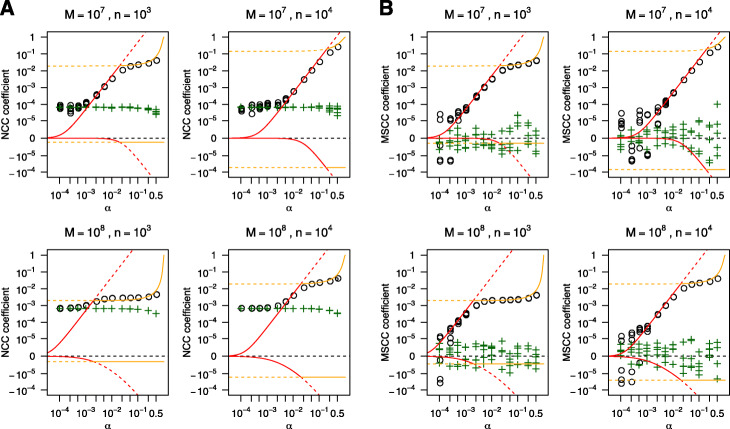
Theoretical predictions and simulation results. Log-modulus plots for (**a**) NCC and (**b**) MSCC comparison between theoretical predictions and simulation results. Red lines indicate the theoretical maximum and minimum in the unsaturated case and orange lines correspond to the saturated case. Circles indicate the calculated maximum coefficients and crosses indicate the minimum coefficients for one out of 5 simulated samples for each parameter combination

To evaluate our results, we generated simulated human ChIP-seq alignments with multiple combinations of parameter values *M*, *n* and *α*. The width *w*, the distance *d* and the sequence read length *R* were fixed as 100, 200 and 50 bp respectively (see Additional file 1 for details). Then NCC and MSCC were calculated from these data and compared to the theoretical predictions (Eqs. (21) and (22), and Eqs. (23) and (24) in Additional file 1, first approximations). As a first evaluation, simulated data were directly generated as aligned read files. The comparison for NCC coefficients demonstrated that our model estimated maximum NCC coefficients with high accuracy for large *α* (Fig. 2a, see Additional file 1: Figure S1 for other combinations of *M* and *n*). In contrast, for minimum NCC coefficients, we observed stable minimum values of coefficients that were almost independent of *α*. Consequently, the maximum NCC coefficient could not be distinguished with its minimum at extremely low *α*. The human genome reference, even the newest assemble, includes some long gaps [30]. These should affect cross-correlation calculations because alignment positions will be biased and the effective genome size becomes shorter than the actual length. The result of the MSCC comparison suggests MSCC solves this issue (Fig. 2b, Additional file 1: Figure S2). These simulations show MSCC maximum coefficients can be detected even if *α* is small (∼ 10^−3^). Therefore, it is expected that MSCC has the advantage of possessing the capacity to assess extremely low S/N samples.

Next, we generated simulated data as substrings of the reference genome sequences and these were mapped back to the reference to investigate effects of the mapping procedure. The stable minimum coefficient values were moderately increased in NCC simulations (Additional file 1: Figure S3). In contrast, in MSCC simulations, we could not find any significant differences from pre-aligned simulations (Additional file 1: Figure S4). These results suggest that not only structural gaps but also genome sequence mappability can affect the calculation of the cross-correlation. Thus, mappability correction is effective in calculating accurate strand cross-correlations.

### Estimating strand cross-correlation in actual data

Our results show that the maximum NCC and MSCC coefficient is a function of the S/N parameter *α*, the total number of binding events *n*, the length of enriched regions *w* and the total number of mapped reads *M* (or *M*_u_). All these parameters can be estimated after peak calling for each ChIP-seq sample. To evaluate whether our model can represent actual ChIP-seq data, we compared true maximum NCC and MSCC coefficients calculated by PyMaSC to coefficients estimated by parameters predicted from peak calling analysis.

In this study, we processed raw sequenced reads with a uniform pipeline which included purifying stpdf to remove duplicated or low mapping quality reads. Therefore, *M* and *M*_u_ can be represented as a total number of mapped reads before and after quality filtering. After peak calling, the total number of called peaks was used as $\hat {n}$. A length of enriched regions *w* was estimated with a shifting model generated by MACS2, densities of reads for each strand in 1,000 highly enriched regions. We calculated *w* as a mean of full width at half maximum (FWHM) of read densities (Additional file 1: Figure S5, see Additional file 1 for details). Lastly, we used FRiP to derive *α*. According to our formulations, FRiP can be written as:

23 $$ \text{FRiP} = \alpha + \frac{n(2w+d)}{G}(1-\alpha)   $$

Therefore, *α* can be estimated by

24 $$ \hat\alpha = \frac{\text{FRiP} - \frac{\hat{n}(2\hat{w} + \hat{d})}{G}}{1 - \frac{\hat{n}(2\hat{w} + \hat{d})}{G}},  $$

where $\hat {w} + \hat {d}$ corresponds to the estimated fragment length. Note that *α* approximates to FRiP when *G* is large enough.

We used the human A549 cell line single-end sequenced ChIP-seq dataset produced by the ENCODE project [5] and the Genomics of Gene Regulation (GGR) project [31] through the ENCODE portal [32]. The dataset included 790 ChIPped samples, which comprised of 59 types of transcription factors (TF), 5 narrow histone marks (H2AFZ, H3K27ac, H3K4me2, H3K4me3 and H3K9ac) and 6 broad histone marks (H3K27me3, H3K36me3, H3K4me1, H3K79me2, H3K9me3 and H4K20me1), and 152 corresponding control samples (see Additional file 2 for dataset details). In the following analyses, we specifically distinguished H3K9me3 because of expected difficulties: H3K9me3 histone marks tend to be enriched in repetitive regions and form megabase scale domains [33], and its antibody, used in ENCODE experiments, could have had a relatively low affinity [34].

**Fig. 3:**
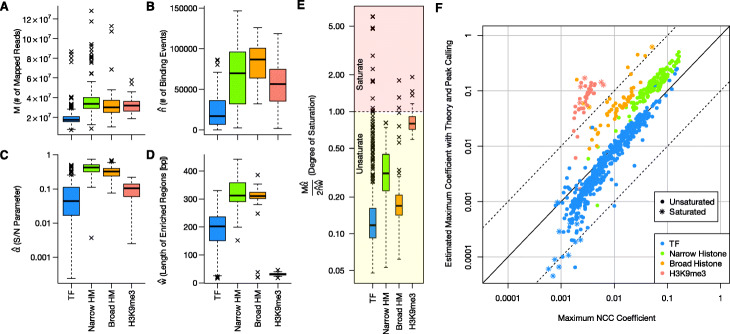
Distributions of parameters and theoretical prediction result for real data. **a**-**d** Distributions of (estimated) parameters *M*, $\hat {n}$, $\hat {\alpha }$ and $\hat {w}$. Note that the y-axis for (C) is logarithmic. **e** Distribution of “degree of saturation” (*Mα*/2*nw*, see Eq. (16)). Where the degree of saturation exceeded one (red area), such samples must be treated as saturated cases. **f** Log-log plot for maximum NCC coefficient comparison between values calculated with PyMaSC (x-axis) and estimated from our model and peak calling analysis (y-axis)

Typically, ChIP targets, which have broader enriched regions or a larger number of binding sites, require a larger number of uniquely mapped reads to call peaks [9]. As recommended in the ENCODE and modENCODE consortia [7], histone mark ChIP-seq samples tend to be sequenced with larger numbers of reads than TF targeting samples (Fig. 3a). Some samples had more than 50 million mapped reads, suggesting that assuming *M*≪*G* could be an over-approximation in several cases. With respect to the numbers of uniquely mapped reads, the number of called peaks $\hat {n}$ and the S/N parameter $\hat {\alpha }$, obtained from FRiP values, clearly differed between TF samples and histone mark samples (Fig. 3b and c). The number of callable peaks and FRiP were highly associated with the number of mapped reads [7]. These tendencies could be seen in our dataset (Additional file 1: Figure S6). Therefore, discussion of how suitable these estimations for the estimation of cross-correlation coefficients are warranted. The distributions $\hat {w}$ demonstrate that estimations for H3K9me3 failed (Fig. 3d). Some plots of shifting models for broad histone marks, especially all H3K9me3 models, demonstrate that there were two characteristic sections in the read density distribution, a sharp peak close to the center and a wide plateau near background levels. Although the second section corresponded to the aspect of the read enriched regions, our method captured only the former sharp peaks (Additional file 1: Figure S7). As a result, estimated width $\hat {w}$ was considerably underestimated for H3K9me3 samples.

To clarify which model can represent typical ChIP-seq situations, we visualised the distributions of “degree of saturation” (Fig. 3e). Only 31 samples out of 790 could be applied to the saturated case. Additionally, saturated TF and H3K9me3 samples were strongly associated with a small number of $\hat {n}$ and/or failure of *w* prediction (Additional file 1: Figure S8). Thus, the unsaturated case will be applicable to most ChIP-seq data. The tendency to relatively high *Mα*/2*nw* values for narrow histone marks reflects their larger number of sequenced reads and higher $\hat {\alpha }$. Broad histone marks, except H3K9me3, had a similar tendency but larger $\hat {n}$ and lower $\hat {\alpha }$ made the tendency weaker.

Although several issues of parameter estimations still remained, estimated maximum coefficients for TF samples were highly consistent with actual maximum NCC coefficients (Fig. 3f). The other types of samples deviated from the centerline; nevertheless, estimated values and actual values exhibited proportionality. This result suggests that the cause of deviation might be systematic errors yielded from the peak calling method, or parameter estimation methods, rather than insufficiency of expressive power of our model. In this analysis we found that not only NCC coefficients but MSCC coefficients were consistent with estimated coefficients (Additional file 1: Figure S9).

### VSN: a novel S/N metric without peak calling

We concluded that the maximum NCC/MSCC coefficient in typical ChIP-seq data is characterised by Eq. (22), where the most interesting parameter is *α*. Thus, the next question is how the formula can be used to derive or assay *α*.

**Fig. 4:**
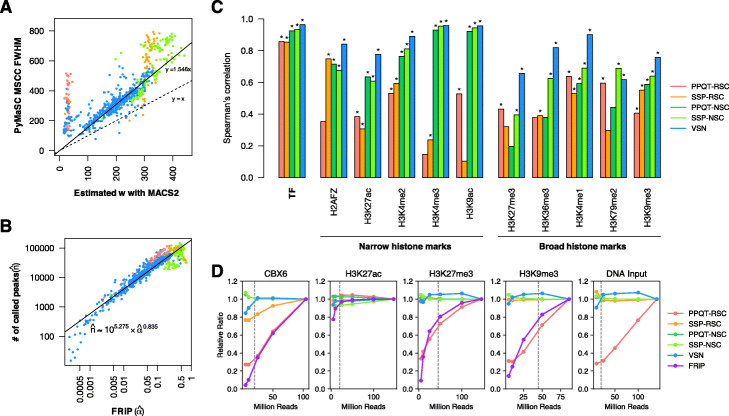
**a** Comparison between the estimated *w* with MACS2 and the FWHM of the PyMaSC MSCC distributions (ENCODE A549 dataset). The solid line shows the result of simple linear regression without the intercept term for the TFs. **b** A log-log plot between the estimated S/N ($\hat {\alpha }$) and the numbers of called peaks ($\hat {n}$) in the ENCODE A549 dataset. **c** Spearman’s correlation between each strand cross-correlation-based metric and $\hat {\alpha }$ (A549 and additional histone-mark dataset). Significant correlations are marked by asterisks (*P*-value < 0.05). **d** Comparison of robustness for sequencing depth. Each sample was down-sampled to 5 M, 10 M, 25 M, 50 M and 100 M (if available) mapped reads. The y-axis is normalised into the relative ratio: for each metric and each sample, scores are normalised against the value at all available mapped reads. Dashed lines indicate the ENCODE criteria of usable reads (20 million for TFs and narrow histone marks and 45 million for broad histone marks)

In Eq. (22), *n*, *w* and *α* are the unknown parameters in the step before peak calling. In the previous section, we estimated *w* from the read enrichment profiles around peaks by using MACS2. On the other hand, strand cross-correlation profiles should have the same information. Ideally, the width of the peak in a strand cross-correlation profile corresponding to the fragment length equals 2*w*, and its FWHM is close to *w*. To check this relation, we observed the distribution of these two evaluations with A549 samples (Fig. 4a). As a result, the values of *w* obtained by these two methods were well proportional in the experiments for TF targets, but there were some significantly different cases, especially those of H3K9me3. To correct these gaps, we performed the simple linear regression of PyMaSC FWHM on MACS2 *w* for TF samples without intercept, and PyMaSC FWHM values were adjusted by its slope to estimate *w*. Then, we calculated the VSN values with the adjusted *w*. To confirm this result from another perspective, we re-estimated the maximum NCC coefficients with *w* values obtained from MSCC distributions instead of the *w* estimated with MACS2, and then, compared the actual maximum coefficients again (Additional file 1: Figure S10). The new *w* values showed significantly decreased deviations for broad histone marks and H3K9me3 samples. Interestingly, TF and narrow histone samples improved in their dispersion and several clusters could be distinguished as lines.

Now, as we have estimated *w* from the cross-correlation distribution, the following relation between *n* and *α* can be obtained and the left side of the equation can be calculated:

25 $$ \frac{\alpha^{2}}{n} \approx \frac{2w}{M} \max{(\text{MSCC})}   $$

Note that a similar equation can be obtained for NCC instead of MSCC, but an advantage of MSCC in *w* estimation is its ability to remove the ‘phantom’ peak [7] because the phantom peak can affect the true peak’s shape [17].

It is expected that a true number of peaks will only depend on biological backgrounds and not on the experimental conditions. However, the actual number of peaks should depend on the noise level, because experiments conducted with lower noise will give better estimation of *n*. If we can assume that the actual number of peaks, which we call $\hat {n}$ in the above text, strongly depends on the noise level, and if we can suppose that $\hat {\alpha }$ is a good indicator of the noise level, we can estimate *α* from Eq. (22). Based on this concept, we named the right side of Eq. (25) VSN (Virtual S/N) and proposed it as a novel metric for ChIP-seq QC assessment:

26 $$ \text{VSN} \coloneqq \frac{2w}{M} \max{(\text{MSCC})}   $$

To evaluate the feasibility of the assumption, we investigated the relation between $\hat {\alpha }$ and $\hat {n}$ by using the experimental data in ENCODE determined for the A549 cell, and found that the order of $\hat {\alpha }$ strongly depended on that of $\hat {n}$ (Fig. 4b).

### Comparison with other cross-correlation based metrics

We compared VSN with other cross-correlation-based metrics and estimated the noise level ($\hat {\alpha }$), NSC and RSC by using an extended A549 dataset (A549 dataset + 182 ChIP-seq data to enlarge histone marks (see Additional file 4 for dataset details), where NSC and RSC were calculated by PPQT [11] and SSP [12] tools, and thus, used two NSCs and RSCs for comparison (these results are available as the Additional file 5). As shown in Figure S11, both PPQT- and SSP-RSC showed moderate correlations with $\hat {\alpha }$ and TFs these metrics have wider dispersion than the others and seem to have weak sensitivity for histone marks. PPQT-NSC, SSP-NSC and VSN showed a similar tendency: clearly lower dispersion than the RSC metrics in both TFs and histone marks. However, while PPQT- and SSP-NSC tended to lose resolution for small values of $\hat {\alpha }$, VSN displayed a consistent relation through a range of $\hat {\alpha }$.

To quantify and compare the correlation between $\hat {\alpha }$ and strand cross-correlation-based metrics, we calculated Spearman’s correlation between FRiP and each metric for TFs and several narrow and broad histone marks (Fig. 4c). PPQT-RSC and SSP-RSC showed low correlations, which agrees with a previously reported result [12]. PPQT-NSC and SSP-NSC indicated higher correlations for TFs and narrow-peak histone marks, but tended to yield low correlations for broad-peak histone marks. In contrast, VSN showed the highest correlations for various ChIP targets except for H3K79me2. We believe that these results support the comprehensive utility of VSN for various ChIP targets and S/N ranges against the pre-existing methods.

Robustness for sequencing depth is a general advantage for strand cross-correlation-based metrics. To validate whether VSN has this nature, compared to the existing ones, we chose five deeply sequenced datasets (ENCFF587OYH for CBX6, ENCFF127TTO for H3K27ac, ENCFF000ALK for H3K27me3,ENCFF000AKK for H3K9me3 and ENCFF000AHV for DNA input) from the A549 dataset and performed down-sampling analysis (Fig. 4d). The results revealed that PPQT-RSC and FRiP are significantly affected by sequencing depth, and the other metrics displayed robustness in contrast. In H3K27ac, exceptionally, there was no remarkable difference. The findings that more than half the peaks called at 100 M reads were found in the 5 M reads in this case, while the others were less than 10%, highlight the dependence of FRiP on the peak calling method. Although PPQT-NSC is robust against sequencing depth, PPQT-RSC is not in this analysis. This result is consistent with the report that duplication removal somewhat reduces the magnitude of phantom peaks [35] as SSP and MSCC apply binary functions to represent mapped reads and duplicated reads are implicitly removed.

### Demonstration of a ChIP-seq qC workflow with VSN

**Fig. 5:**
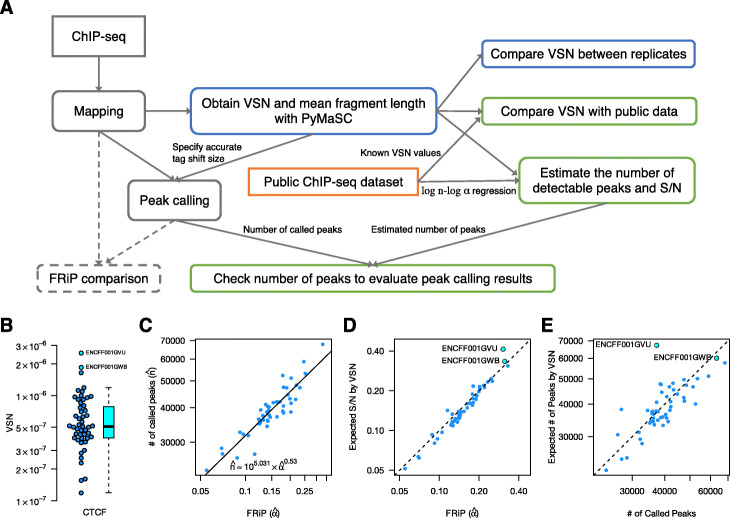
ChIP-seq QC workflow using VSN and its demonstration. **a** A ChIP-seq QC workflow using VSN. VSN can be obtained before peak calling and can be compared between replicates as S/N (blue boxes). If VSN values for public data are available, experimentalists can rank their samples against previous experiments. Moreover, *n*- *α* regression enables the estimation of the expected number of peaks and S/N separately before peak calling (green boxes). **b** Example of VSN distribution. **c** Example of *n*- *α* regression. **d** Comparison between actual FRiP values and S/Rs estimated by VSN and the regression equation. **e** Comparison between the actual number of called peaks and the expected number of peaks

Until here, we flocculated ChIP-seq data analyses and proposed a novel QC metric, called VSN, and here, we would like to propose a new ChIP-seq QC workflow as a VSN application (Fig. 5a). As a demonstration, we showed how VSN analysis can be applied in the case of the ENCODE A549 CTCF dataset (see Additional file 1 for details). The insulator binding factor CTCF (CCCTC-binding factor) is known as a highly conserved transcription factor [36].

Let us assume that we have multiple ChIP-seq experiments for a ChIP target. Just after mapping, we can calculate VSN values for these samples. In Fig. 5b, the VSN distribution for 49 CTCF ChIP-seq samples from 19 experiments is shown. Based on such observation, a VSN threshold can be established for a ChIP target (e.g. 2×10^−7^ or 3×10^−7^ in this case). Although this strategy can also be performed using FRiP, quality assessment using VSN values is no longer biased towards any difference in the peak calling methods.

As described, VSN can be used to evaluate the quality of experimental data before peak calling. In addition, it can be used to evaluate the quality of peak calling methods by using a set of experimental data. Now, we have considerable experimental data in public databases, as in the case of the ENCODE project. Thus, we can see the distribution of *α* and *n* with a peak calling method by using a set of experimental data. By using the distribution, we can create a regression equation between them (ex. Fig. 5c). On the other hand, for a single ChIP-seq dataset, we can calculate VSN that is equivalent to *α*^2^/*n* according to our formulation, which gives an equation between $\hat {\alpha }$ and $\hat {n}$ besides the regression equation. Therefore, by combining these two equations, we can calculate $\hat {\alpha }$ and $\hat {n}$, and compare them with the results of the peak calling (ex. Fig. 5d and e). This comparison gives us new insights into the quality of the peak calling methods, as demonstrated in the case of CTCF.

Let us consider that an experiment ENCSR000DPF, which has two biological replicates ENCFF001GWB and ENCFF001GVU, is newly performed, and all other 28 CTCF ChIP-seq experiments are publicly available. First, we can calculate and compare VSN values between the public data and newly obtained data. As shown in Fig. 5b, both replicates have extremely high VSN values, and we can expect these samples to indicate high FRiP scores and yield several called peaks in this phase. Second, by using the peak calling flow described in Methods, we can obtain a scatter plot between $\hat {\alpha }$ and the number of called peaks ($\hat {n}$) for public experiments (Fig. 5c), which gives a linear regression equation shown in Fig. 5c, by using the lm function in R. Third, by using this equation and VSN, we calculate $\hat {\alpha }$ and $\hat {n}$, and refer to them as “Expected S/N by VSN” and “Expected number of Peaks by VSN,” respectively. At the same time, we perform peak calling, obtain the actual number of called peaks, and calculate the FRiP scores. Finally, we compare FRiP with the expected S/N (Fig. 5d) and the number of called peaks with the expected number of peaks (Fig. 5e).

In the comparison of the noise level (*α*), ENCFF001GVU’s FRiP value was unexpectedly much smaller than ENCFF001GWB’s, against the rank of VSN and expected S/N. This deviation was more remarkable in the comparison for the number of peaks: we expected that about 67,000 peaks would be called for ENCFF001GVU. In fact, 63,041 peaks were called for the other replicate. However, only 37,116 peaks were detected in the actual analysis. Because this result implies failure in the peak calling flow, we investigated each analysis step and found that 65% reads were detected as duplicate and only 2.2 M were available for peak calling in ENCFF001GVU (vs 19% and 10.7 M reads in ENCFF001GWB, respectively). The small read number caused insufficient peak calling, and consequently, FRiP provided an underestimated S/N score. In contrast, the better VSN value for ENCFF001GVU, which reflects its sufficient ChIP enrichment, suggests that the data can be used for peak calling by merging other data, for example, replicates like ENCFF001GWB. These results demonstrate that a combination of publicly available data and our VSN formulation enables us to assess ChIP enrichment and the quality of the peak calling methods independently.

### Limitation of normalised FRiP

Through this study, we had focused on FRiP instead of normalised FRiP, because peak calling with a reduced number of mapped reads could cause inaccurate peak calling. As a result, normalised FRiP can be problematic as the S/N indicator. In the calculation of normalised FRiP, we need to determine the number of reads for normalisation. We chose 10 M mapped reads as the normalisation criterion based on the distribution of the number of available mapped reads (Additional file 1: Figure S12). This threshold is much higher than the pre-existing pipeline (4 M mapped reads)[8]. It should be noted the larger number of reads used for normalisation is better for peak call, but too much larger values resulted in the smaller number of available samples.

We observed the changes in FRiP values and the number of called peaks at the 10 M reads against their original values (Additional file 1: Figure S13). It is expected that FRiP value will decrease as shown in Figure. 4D, and that the number of called peaks will decrease as mapped reads are reduced because weak peaks will disappear. However, we unexpectedly observed that the numbers of called peaks were not significantly decreased, while normalised FRiP values became much smaller (∼ 1/10th) than the original FRiP. The result raises two possibilities: (1) some peaks were called as false positives in a normalised dataset, or (2) most peaks were truly detected but their genomic ranges were shortened, and thus many signal reads were not included on the FRiP calculation. To clarify which scenario was actually happened, we plotted recall and precision based on the total length of peaks against original peak calling results and observed that most of the samples retained high precision and divergent recall (Additional file 1: Figure S14). High precision values in most samples suggest false positives are unlikely occurred in the down-sampling analysis, but various recall values indicate the change of peak widths at the reduced depth.

According to these results, the second scenario is more likely than the first possibility. Therefore, normalised FRiP can be easily unreliable.

## Discussion

We demonstrated that our ChIP-seq read density model can be applied for the theoretical prediction of NCC and MSCC coefficients. However, our model has some limitations in expressing details of the actual ChIPseq data. For example, *w* and *d* should have distributions, or more fundamentally, the probability of a read mapping around a binding site should be more complex than the enriched region model with a uniform probability. More detailed modelling and simulation may reveal the detailed nature of strand cross-correlation profiles. Nevertheless, detailed modelling of the probability distribution is not straightforward as non-uniform distribution is the outcome of DNA fragmentation, i.e. sonication biases caused by binding elements and nucleotide sequences. Considering our consistent result with the real data, we expect our analysis to have captured the essence of strand cross-correlation profiling, at least as a first approximation.

In previous studies, the NSC, a ratio of a maximum and a minimum coefficient, was proposed as a metric to evaluate the S/N in ChIP-seq. However, according to our derivation, the minimum coefficient should be a negative value and nearly zero. This result suggests that VSN will be more informative than NSC. In contrast, read densities could be affected by various technical or biological biases [3] and actual strand cross-correlation profiles can continue to decline at shift sizes exceeding 10 kb and the background level of strand cross-correlation profiles could become higher than the ideal uniform distribution [12]. Therefore, we cannot deny the NSC’s potential to be normalised by semi-background levels, implying the size of the total number of reads, or reflecting the background uniformity of ChIP-seq data. Furthermore, since we characterised only NCC and MSCC in this study, further approaches to theoretical characterisation and computational comparisons will be needed to discuss differences between strand cross-correlation profiling methods.

The errors between the actual and estimated NCC coefficients observed in Fig. 3f, as well as those still observed after *w* corrections (Additional file 1: Figure S10), could imply insufficiency of our modelling. However, imperfect peak calling and consequential parameter estimation can also cause these errors. According to Eq. (22), underestimation for *n* or overestimation for *α* causes overestimation of the maximum coefficient. Although both the number of called peaks and the enriched region length in histone marks were about two times larger than the TFs in our dataset (see Figs. 3b, d and 4b), we found that the total length of genomic regions, called peaks, was about ten times longer (Additional file 1: Figure S15). This result could be explained if multiple binding events were called as a single peak, or the peak width was overestimated due to the peak caller’s limitation. As a result, *n* can be underestimated or *α* can be overestimated. These findings can support the difficulty of accurate FRiP calculation and its utilisation.

## Conclusions

QC methods for ChIP-seq based on strand cross-correlation have relied on uncorroborated metrics and empirically determined thresholds. As a result, strand cross-correlation based approaches are vague for QC metrics in terms of what they actually measure, even though real analysis results support the correlation between the metrics and ChIP quality, and their potential and limitations have also been unclear. In this study, we derived the theoretical minimum and maximum of NCC and MSCC coefficients using the ChIP-seq read density model which has the S/N parameter. The results revealed that maximum of NCC and MSCC coefficients can be regard as the function of the total number of mapped reads, the enriched region length, the total number of binding events and the S/N parameter. The relation clarifies how the coefficients can be comparable and implies the limitation for estimating the total number of binding events and S/N separately within the strand cross-correlation analysis. Based on these insights, we proposed VSN, a novel S/N metric that does not require peak calling and consistently estimates S/N for various ChIP targets and sequencing read depths. Furthermore, we demonstrated that VSN analysis combined with pre-existing peak calling results enables to assess ChIP enrichment and the quality of the peak calling methods independently. Our study provides the first theoretical insight into the strand cross-correlation and will strongly support ChIP-seq QC and the establishment of better peak calling methods.

## Supplementary information

**Additional file 1** This file contains all supplementary methods and figures.

**Additional file 2** The list of experiments in ENCODE and GRR project used in this study.

**Additional file 3** The R script to calculate the enriched region length from a peak model generated by MACS2. See Additional file 1 for details.

**Additional file 4** The list of additionally analysed ChIP-seq experiments obtained from ENCODE and Roadmap project.

**Additional file 5** ChIP-seq analysis results of the strand cross-correlation based metrics and peak calling.

## Data Availability

PyMaSC is freely available as a Python package and the source code can be found in https://github.com/ronin-gw/PyMaSC. All mappability tracks used in this study are available at https://pymasc.sb.ecei.tohoku.ac.jp. Implementation of the ChIP-seq read density simulation based on our model is available at https://github.com/ronin-gw/chipseq-simdata-generator. We used real ChIP-seq data obtained from the ENCODE Portal https://www.encodeproject.org/. Other materials and methods are available as Additional files.

## Abbreviations

CTCF: CCCTC-binding factor
ChIP: Chromatin immunoprecipitation
ChIP-seq: Chromatin immunoprecipitation followed by sequencing
FRiP: Fraction reads in peaks
FWHM: Full width at half maximum
HM: Histone mark
MSCC: Mappability-sensitive cross-correlation
NCC: Naïve cross-correlation
NGS: Next-generation sequencing
NSC: Normalised strand coefficient
PPQT: phantompeakqualtools
QC: Quality control
RSC: Relative strand correlation
S/N: Signal-to-noise ratio
SSP: Strand-shift profile
TF: Transcription factor
VSN: Virtual S/N
