# Supplementary material for: Theoretical characterisation of strand cross-correlation in ChIP-seq
Source: BMC Bioinformatics. 2020 Sep 22;21:417. doi: 10.1186/s12859-020-03729-6 (PMC7510163; doi:10.1186/s12859-020-03729-6)
Supplement: Supplementary file 1 — Additional file 1 This file contains all supplementary methods and figures. [file 12859_2020_3729_MOESM1_ESM.docx]

Theoretical characterisation of
strand cross-correlation in ChIP-seq

Hayato Anzawa, Hitoshi Yamagata and Kengo Kinoshita

# Supplementary Material Table of Contents

1. Settings for the Saturated Case
2. Expected Value of MSCC Coefficients
3. Complete Formulas for Maximum and Minimum in Saturated Cases
4. Simulation ChIP-seq Read and Alignment Data Generation Procedure
5. Supplementary Figures
   - Figure S1. Log-modulus plots for NCC comparison between the theoretical prediction and calculated from the simulated data without mapping for all parameter combinations.
   - Figure S2. Log-modulus plots for MSCC comparison between the theoretical prediction and calculated from the simulated data without mapping for all parameter combinations.
   - Figure S3. Log-modulus plots for NCC comparison between the theoretical prediction and calculated from the simulated data with mapping.
   - Figure S4. Log-modulus plots for MSCC comparison between the theoretical prediction and calculated from the simulated data with mapping.
   - Figure S5. An example of MACS2 shifting model and its width detection result.
   - Figure S6. Pair plots for estimated parameters.
   - Figure S7. An example of MACS2 shifting model for a H3K9me3 sample and its width detection result.
   - Figure S8. Relation between $M\alpha/2nw$ and estimated parameters.
   - Figure S9. Log-log plot for maximum MSCC coefficient comparison.
   - Figure S10. Log-log plot for maximum NCC coefficient comparison after $w$ correction.
   - Figure S11. Log-log plot for comparison between S/N parameter $\hat{\alpha}$ and strand cross-correlation based metrics.
   - Figure S12. Histogram of the number of mapped reads on the entire ENCODE dataset.
   - Figure S13. Comparison in the number of called peaks and FRiP values between before and after normalisation.
   - Figure S14. Precision-recall plot of the peak calling results at 10 M reads.
   - Figure S15. Distributions of the total length of genomic regions called as peaks.
   - Figure S16. Comparison between maximum NCC and MSCC coefficients.

**Setting for the saturated case**

For saturated cases, we assumed that (1) all the enriched regions were filled by mapped reads; and (2) the effective size of $M$ will be smaller since duplicated reads in enriched regions are not contained in $f$ and $g$. Specifically, (1) $p_{S}=1$ and (2) $M$ must be replaced with $M_{u}$ when $\mu$ is calculated. Finally, the settings for the saturated cases are:

$$\begin{aligned} \text{Saturated Case}:=\left\{ \begin{aligned} \mu=\frac{M_{u}}{2G} \\ p_{S}=1 \\ p_{N}=\frac{M}{2G}\left( 1-\alpha\right) \end{aligned} \right. \#\left( 1 \right) \end{aligned}$$

**Expected value of MSCC coefficient**

The MSCC of $f$ and $g$ at a shift size $x$ is formally written and approximately derived as follows (Ramachandran, *et al.*, 2013):

$$\begin{aligned} \text{MSCC}\left( f,g \right)\left( x \right)= \frac{\frac{1}{\left| U^{x} \right|}\left( \sum_{i\in U^{x}} (f\left( i \right)g(i+x) \right)-\mu_{f}^{x}\mu_{g}^{x}}{\sqrt{\sigma_{f}^{x}\sigma_{g}^{x}}} \#\left( 2 \right) \end{aligned}$$

where $U^{x}$ is the set of the doubly mappable positions when the shift size is $x$. The $\mu_{f}^{x}$, $\mu_{g}^{x}$, $\sigma_{f}^{x}$ and $\sigma_{g}^{x}$ are the means and variances for $f$ and $g$ corresponding to the $U^{x}$: $\mu_{f}^{x}=\sum_{i\in U^{x}} f\left( i \right)/|U^{x}|$, $\mu_{g}^{x}=\sum_{i\in U^{x}} g\left( i \right)/|U^{x}|$, $\sigma_{f}^{x}=\mu_{f}^{x}(1-\mu_{f}^{x})$ and $\sigma_{g}^{x}=\mu_{g}^{x}(1-\mu_{g}^{x})$. In this section, we derive the expected value of MSCC following the same method as described in the main manuscript.

First, we define “doubly mappable ratio”, the ratio of the total length of doubly mappable regions to the genome length:

$$\begin{aligned} \beta:=\frac{\left| U^{x} \right|}{G} \#\left( 3 \right) \end{aligned}$$

Note that $0\leq\beta\leq1$ since $U^{x}$ is a subset of genomic positions. Second, we assumed that mapped reads are uniformly distributed among the genome independently of mappability. Based on our model, the numbers of reads mapped into $U^{x}$ of the forward- and reverse-strand are:

$$\begin{aligned} \sum_{i\in U^{x}} f\left( i \right)=\sum_{i\in U^{x}} g\left( i \right)=\frac{M}{2}\beta\#\left( 4 \right) \end{aligned}$$

Similarly, we assumed both forward- and reverse-enriched regions are uniformly distributed. More formally, we assumed that only $n\beta$ binding sites belong to $U^{x}$ and let $E_{f}^{x}$ and $E_{g}^{x}$ as the intersections of enriched regions and $U^{x}$:

$$\begin{aligned} n^{x}:=n\beta\#\left( 5 \right) \end{aligned}$$

$$\begin{aligned} \frac{\sum_{i\in E_{f}^{x}} f\left( i \right)}{\sum_{i\in E_{f}} f\left( i \right)}=\frac{\sum_{i\in E_{g}^{x}} g\left( i \right)}{\sum_{i\in E_{g}} g\left( i \right)}=\beta\#(6) \end{aligned}$$

where $E_{f}^{x}:=E_{f}\cap U^{x}$ and $E_{g}^{x}:=E_{g}\cap U^{x}.$ Therefore, total numbers of signal and noise reads mapped into the $U^{x}$ can be written as:

$$\begin{aligned} \sum_{i\in E_{f}^{x}} f\left( i \right)=\sum_{i\in E_{g}^{x}} g\left( i \right)=\frac{M}{2}\alpha\beta\#(7) \end{aligned}$$

$$\begin{aligned} \sum_{i\in B_{f}^{x}} f\left( i \right)=\sum_{i\in B_{g}^{x}} g\left( i \right)=\frac{M}{2}\left( 1-\alpha\right)\beta\#\left( 8 \right) \end{aligned}$$

where $B_{f}^{x}=\left\{ i | \left( i\notin E_{f}^{x} \right)\bigwedge(i\in U^{x}) \right\}$ and $B_{g}^{x}=\left\{ i | \left( i\notin E_{g}^{x} \right)\bigwedge(i\in U^{x}) \right\}$.

According to Equation (2) and (3), $\mu_{f}^{x}$ and $\mu_{g}^{x}$ can be calculated as:

$$\begin{aligned} \mu_{f}^{x}=\frac{\sum_{i\in U^{x}} f\left( i \right)}{\left| U^{x} \right|}=\frac{M\beta/2}{G\beta}=\frac{M}{2G}=\mu\#\left( 9 \right) \end{aligned}$$

$$\begin{aligned} \mu_{g}^{x}=\frac{\sum_{i\in U^{x}} g\left( i \right)}{\left| U^{x} \right|}=\frac{M\beta/2}{G\beta}=\frac{M}{2G}=\mu\#(10) \end{aligned}$$

Thus, $\mu_{f}^{x}=\mu_{g}^{x}=\mu$ and $\sigma_{f}^{x}=\sigma_{g}^{x}=\sigma$. In this step, Equation (1) can be rewritten as:

$$\begin{aligned} \text{MSCC}\left( f,g \right)\left( x \right)=\frac{1}{\sigma}\left( \frac{\sum_{i\in U^{x}} f\left( i \right)g\left( i+x \right)}{G\beta-x}-\mu^{2} \right) \#\left( 11 \right) \end{aligned}$$

Therefore, MSCC can be estimated as:

$$\begin{aligned} \left\langle MSCC\left( f,g \right)\left( x \right) \right\rangle=\frac{1}{\sigma}\left( \frac{\left\langle\left| D_{U^{x}} \right| \right\rangle}{G\beta-x}-\mu^{2} \right) \#\left( 12 \right) \end{aligned}$$

where $|D_{U^{x}}|$ is the total count of the doubly mappable positions $i$ which give both $f\left( i \right)=1$ and $g\left( i+x \right)=1$.

To estimate $D_{U^{x}}$, we prepared the probabilities that $f$ becomes 1 at a position $i$ within $U^{x}$ ($P_{f=1}^{x}(i)$) and $g$ becomes 1 ($P_{g=1}^{x}(i)$). As described in the main manuscript, the expected value of $|D_{U^{x}}|$ can be described as:

$$\begin{aligned} \left\langle\left| D_{U^{x}} \right| \right\rangle=\sum_{i\in U^{x}} 1\cdot P_{f=1}^{x}\left( i \right)P_{g=1}^{x}\left( i+x \right) \#\left( 13 \right) \end{aligned}$$

In more detail, $P_{f=1}^{x}$ can be written using the probabilities to observe a signal read at a forward position $i$ within the doubly mappable positions ($P_{S,f=1}^{x}$) and to observe a noise read ($P_{N,g=1}^{x}$):

$$P_{f=1}^{x}\left( i \right):=1-\left( 1-P_{S,f=1}^{x}(i) \right)\left( 1-P_{N,g=1}^{x}(i) \right)$$

$$\begin{aligned} =\left\{ \begin{aligned} p_{S}^{x} &\text{if} i\in E_{f}^{x} \\ p_{N}^{x} &\text{if} i\notin E_{f}^{x} \end{aligned} \right. \#\left( 14 \right) \end{aligned}$$

The similar result can be obtained for $P_{g=1}^{x}\left( i \right)$:

$$\begin{aligned} P_{g=1}^{x}\left( i \right)=\left\{ \begin{aligned} p_{S}^{x} &\text{if} i\in E_{g}^{x} \\ p_{N}^{x} &\text{if} i\notin E_{g}^{x} \end{aligned} \right. \#\left( 15 \right) \end{aligned}$$

Thus, $P_{f=1}^{x}\left( i \right)P_{g=1}^{x}\left( i+x \right)$ can be expanded as:

$$\begin{aligned} P_{f=1}^{x}\left( i \right)P_{g=1}^{x}\left( i+x \right)=\left\{ \begin{aligned} \left( p_{S}^{x} \right)^{2} &\text{if} i\in X_{SS}^{x} \\ p_{S}^{x}p_{N}^{x} &\text{if} i\in X_{SN}^{x} \\ p_{N}^{x}p_{S}^{x} &\text{if} i\in X_{NS}^{x} \\ \left( p_{N}^{x} \right)^{2} &\text{if} i\in X_{NN}^{x} \end{aligned} \right. \#(16) \end{aligned}$$

where

$$\begin{aligned} \begin{matrix} \begin{matrix} X_{SS}^{x}:=\left\{ i | \left( i\in E_{f}^{x} \right)\bigwedge\left( i+x\in E_{g}^{x} \right) \right\} \\ X_{SN}^{x}:=\left\{ i | \left( i\in E_{f}^{x} \right)\bigwedge\left( i+x\in B_{g}^{x} \right) \right\} \end{matrix} \\ \begin{matrix} X_{NS}^{x}:=\left\{ i | \left( i\in B_{f}^{x} \right)\bigwedge\left( i+x\in E_{g}^{x} \right) \right\} \\ X_{NN}^{x}:=\left\{ i | \left( i\in B_{f}^{x} \right)\bigwedge\left( i+x\in B_{g}^{x} \right) \right\} \end{matrix} \end{matrix} \#\left( 17 \right) \end{aligned}$$

We assumed the sizes of these sets approximately equal $\beta$ times the sizes of sets defined in Equation (14) in the main manuscript:

$$\begin{aligned} \begin{matrix} \begin{matrix} \left| X_{SS}^{x} \right|\approx\beta\left| X_{SS} \right| \\ \left| X_{SN}^{x} \right|\approx\beta\left| X_{SN} \right| \end{matrix} \\ \begin{matrix} \left| X_{NS}^{x} \right|\approx\beta\left| X_{NS} \right| \\ \left| X_{NN}^{x} \right|\approx\beta\left| X_{NN} \right| \end{matrix} \end{matrix} \#\left( 18 \right) \end{aligned}$$

Therefore, $\left\langle\left| D_{U^{x}} \right| \right\rangle$ can be obtained as:

$$\begin{aligned} \begin{matrix} \left\langle\left| D_{U^{x}} \right| \right\rangle=\left| X_{SS}^{x} \right|\left( p_{S}^{x} \right)^{2}+\left( \left| X_{SN}^{x} \right|+\left| X_{NS}^{x} \right| \right)p_{S}^{x}p_{N}^{x}+\left| X_{NN}^{x} \right|\left( p_{N}^{x} \right)^{2} \\ \approx\beta\left( \left| X_{SS} \right|\left( p_{S}^{x} \right)^{2}+\left( \left| X_{SN} \right|+\left| X_{NS} \right| \right)p_{S}^{x}p_{N}^{x}+\left| X_{NN} \right|\left( p_{N}^{x} \right)^{2} \right) \end{matrix}\#\left( 19 \right) \end{aligned}$$

In the unsaturated cases, $P_{S,f=1}^{x}$ and $P_{N,f=1}^{x}$ can be expanded as:

$$\begin{aligned} \begin{matrix} P_{S,f=1}^{x}\left( i \right)=\left\{ \begin{aligned} \frac{\sum_{i\in E_{f}^{x}} f\left( i \right)}{n^{x}w}=\frac{\frac{M\alpha\beta}{2}}{n\beta w}=\frac{M}{2nw}\alpha&\text{if} i\in E_{f}^{x} \\ 0 &\text{if} i\notin E_{f}^{x} \end{aligned} \right. \\ P_{N,f=1}^{x}\left( i \right)=\frac{\sum_{i\in B_{f}^{x}} f\left( i \right)}{\left| U^{x} \right|}=\frac{M\left( 1-\alpha\right)\frac{\beta}{2}}{G\beta}=\frac{M}{2G}\left( 1-\alpha\right) \end{matrix} \#\left( 20 \right) \end{aligned}$$

The probabilities are the same as the NCC derivation (Equation (9) and (10) in the main manuscript). The same result can be obtained for $P_{S,g=1}^{x}$ and $P_{N,g=1}^{x}$. These results mean $p_{S}^{x}=p_{S}$ and $p_{N}^{x}=p_{N}$ (Equation (18) in the main manuscript). In the saturated cases, the same assumptions can be applied as were used in the derivation of the NCC saturated case: (i) $p_{S}^{x}=1$ and (ii) $M_{u}$ must be used instead of $M$ when $\mu$ is calculated. As a result, $p_{S}^{x}=p_{S}$ and $p_{N}^{x}=p_{N}$ are similarly formed.

Finally, for both unsaturated and saturated cases, Equation (18) can be rewritten as:

$$\begin{aligned} \left\langle\left| D_{U^{x}} \right| \right\rangle\approx\beta\left( \left| X_{SS} \right|p_{S}^{2}+\left( \left| X_{SN} \right|+\left| X_{NS} \right| \right)p_{S}p_{N}+\left| X_{NN} \right|p_{N}^{2} \right)=\beta\left\langle\left| D_{x} \right| \right\rangle\#\left( 21 \right) \end{aligned}$$

and Equation (11) becomes:

$$\begin{aligned} \begin{matrix} \left\langle MSCC\left( f,g \right)\left( x \right) \right\rangle\approx\frac{1}{\sigma}\left( \frac{\beta\left\langle\left| D_{x} \right| \right\rangle}{G\beta-x}-\mu^{2} \right) \\ \approx\frac{1}{\sigma}\left( \frac{\left\langle\left| D_{x} \right| \right\rangle}{G}-\mu^{2} \right) \\ \approx\left\langle NCC\left( f,g \right)\left( x \right) \right\rangle\end{matrix} \#\left( 22 \right) \end{aligned}$$

The second approximation assumes $G\beta\gg x$ and the third approximation means $G\gg x$. The conclusion means it is expected that both NCC and MSCC give almost the same coefficient. In our test dataset, observed maximum NCC and MSCC coefficients were very similar (Supplementary Figure S16).

**Complete formulas for minimum and maximum values in saturated cases**

For saturated cases, the minimum and maximum value can be calculated the same as the unsaturated cases using settings shown in Equation (1). The minimum is:

$$\text{NCC}\left( f,g \right)\left( x_{\text{far}} \right)=-\frac{1}{G-x_{\text{far}}}\frac{2nw}{M_{\text{N}}+2nw}\frac{M_{\text{N}}^{2}-2M_{\text{N}}x_{\text{far}}+2nw\left( G-x_{\text{far}} \right)}{2G-\left( M_{\text{N}}+2nw \right)}$$

$$\begin{aligned} \approx-\frac{1}{G}\frac{2nw}{M_{\text{N}}+2nw}\frac{2Gnw+M_{\text{N}}^{2}}{2G} \#\left( 23 \right) \end{aligned}$$

$$\approx0$$

where $M_{\text{N}}=\left( 1-\alpha\right)M_{u}$. And the maximum is:

$$\text{NCC}\left( f,g \right)\left( d+w \right)=\frac{1}{G-\left( d+w \right)}\frac{2nw}{M_{\text{N}}+2nw}\frac{2\left( G-\frac{M_{\text{N}}}{2} \right)^{2}-\left( M_{\text{N}}^{2}-2M_{\text{N}}\left( d+w \right)+2nw\left( G-\left( d+w \right) \right) \right)}{2G-\left( M_{\text{N}}+2nw \right)}$$

$$\begin{aligned} \approx\frac{1}{G}\frac{2nw}{M_{\text{N}}+2nw}\frac{2\left( G-\frac{M_{\text{N}}}{2} \right)^{2}-\left( M_{\text{N}}^{2}-2M_{\text{N}}\left( d+w \right)+2nwG \right)}{2G-\left( M_{\text{N}}+2nw \right)} \#\left( 24 \right) \end{aligned}$$

$$\approx\frac{2nw}{M_{\text{N}}+2nw}=\frac{nw}{\frac{M_{u}}{2}\left( 1-\alpha\right)+nw}$$

**Procedure for generation of simulation ChIP-seq read and alignment data**

Here we describe the procedure to generate ChIP-seq simulation data from a reference genome FASTA file with the following parameters:

- $n$: Total number of binding events
- $w$: Width of enriched regions [bp]
- $M$: Total number of (mapped) reads
- $\alpha$: Signal and noise mixture parameter ($0 \leq\alpha\leq1$)
- $d$: Distance between forward and reverse enriched regions [bp]
- $l$: Sequence reads’ length [bp]+

The implementation used in this study is available at <https://github.com/ronin-gw/chipseq-simdata-generator>.

1. A given FASTA file is parsed and lengths and sequences for each chromosome are stored. Alternate or unlocalised contigs were ignored in this study. Note that ‘N’ bases are not included in counts and substrings that include ‘N’ are not chosen in the later generation step.
2. The number of binding events for each chromosome is randomly chosen according to the relative weights based on each chromosome length.
3. For each chromosome, peak regions, having a length $2w+d$, are chosen from its entire sequence. If a chosen region overlaps with a region already chosen, or chosen sequence includes ‘N’, the sampling is retried. These steps are iterated until the determined number of peak regions has been chosen.
4. Similar to step (2), the numbers of reads are assigned for each chromosome. For each chromosome, random reads and signal reads are generated with a ratio of $\left( 1-\alpha\right):\alpha$.
5. Random reads, the total number is $M(1-\alpha)$, are generated as follows: For each chromosome, sequences with length $2w+d$ bp are sampled from the entire sequence of the chromosome. If a sampled sequence includes ‘N’, the sequence is discarded and resampling is not performed. A read is generated as the reverse read with the probability of 1/2. When a read is generated as a forward read, a read start position is randomly selected from the beginning of $w$ bp of the sequence, and a read is picked out as a substring from the chosen start position to $l$ bp ahead. When a read is generated as a reverse read, a read start position is randomly selected from the end of $w$ bp of the sequence, and a read is picked out as the reverse-complement of a substring from the chosen start position to $l$ bp behind.
6. As the same as step (5), signal reads, the total number is $M\alpha$, are generated by randomly choosing peak regions instead of choosing fragments uniformly from the whole chromosome.
7. The generated reads are written as FASTQ or SAM format. All sequencing quality (Phred) scores are outputted as 40 (‘I’).

In this study, we obtained 5 iterations of simulation data with the following parameter combinations:

- $n=\{100, 1000, 10000, 100000\}$
- $w=100$
- $M=\{10\times{10}^{6}, 50\times{10}^{6}, 100\times{10}^{6}\}$
- $\alpha=\{0.0001, 0.00025, 0.0005, 0.001, 0.0025 ,0.005, 0.01, 0.025, 0.05, 0.1, 0.25, 0.5\}$
- $d=200$
- $l=50$

**Enriched region length estimation with a MACS2 peak model**

We used the R script attached as Additional file 3 to obtain the enriched region length from a peak model generated by MACS2. The script must be concatenated with a **_model.r* file generated by MACS2 for plotting a peak model. Note that we used a smaller median value from first or last 50 bp as a minimum value because the values of each edge can be suddenly decreased (see Supplementary Figure S7).

Figure S1. Log-modulus plots for NCC comparison between the theoretical prediction and calculated from the simulated data without mapping for all parameter combinations.

Figure S2. Log-modulus plots for MSCC comparison between the theoretical prediction and calculated from the simulated data without mapping for all parameter combinations.

Figure S3. Log-modulus plots for NCC comparison between the theoretical prediction and calculated from the simulated data with mapping.

Figure S4. Log-modulus plots for MSCC comparison between the theoretical prediction and calculated from the simulated data with mapping.

Figure S5. An example of MACS2 shifting model (left) and its width detection result (right). Red and green dash lines in the right plot show the half heights of forward and reverse peaks. The full width at half maximum (FWHM) of forward and reverse peaks are indicated as red and blue numbers and their mean is indicated at the bottom centre of the box.

Figure S6. Pair plots for estimated parameters $\boldsymbol{M,}\hat{\boldsymbol{n}}\boldsymbol{,}\hat{\boldsymbol{\alpha}}$ and $\hat{\boldsymbol{w}}$. A strong positive correlation was observed between $\hat{\boldsymbol{n}}$ and $\hat{\boldsymbol{\alpha}}$. There were also weak positive relations between $\boldsymbol{M}$ and $\hat{\boldsymbol{n}}$, and between $\boldsymbol{M}$ and $\hat{\boldsymbol{\alpha}}$.

Figure S7. An example of MACS2 shifting model for an H3K9me3 sample and its width detection result. There were two levels of the peak height and the plot demonstrates that our method detected only higher sharp peak as the enriched region.

Figure S8. The relation between $\boldsymbol{M\alpha/2}\boldsymbol{nw}$ (degree of saturation) and estimated parameters: (A) number of called peaks, (B) width of enriched regions, (C) number of mapped reads and (D) signal noise mixture parameter. Blue, green, orange and pink plots indicate ChIP targets (transcription factors (TF), narrow histone modifications, broad histone modifications and H3K9me3, respectively). TF samples whose $\boldsymbol{M\alpha/2}\boldsymbol{nw}$ exceeded 1 were related to low numbers of called peaks, short detected length of enriched regions and low $\hat{\boldsymbol{\alpha}}$ suggesting that poor enrichment and consequential failure of $\boldsymbol{w}$ estimation make $\boldsymbol{M\alpha/2}\boldsymbol{nw}$ higher.

Figure S9. Log-log plot for maximum MSCC coefficient comparison.

Figure S10. Log-log plot for maximum NCC coefficient comparison after $\boldsymbol{w}$ correction.

Figure S11. Log-log plot for comparison between S/N parameter $\hat{\boldsymbol{\alpha}}$ and strand cross-correlation based metrics.

Figure S12. Histogram of the number of mapped reads on the entire ENCODE dataset. The vertical red line indicates a chosen threshold (10 M mapped reads).

Figure S13. Comparison in the number of called peaks and FRiP values between before and after normalisation. The black lines indicate y=x. Left panel: comparison between the original number of called peaks and the number of peaks at 10 M reads. Right panel: comparison between the FRiP values and the normalised FRiP values.

Figure S14. Precision-recall plot of the peak calling results at 10 M reads. These metrics were calculated based on the total length of the genomic regions called as peaks.

Figure S15. Distributions of the total length of genomic regions called as peaks.

Figure S16. Comparison between maximum NCC and MSCC coefficients.
